# Designing enhanced mixing in stagnant microfluidic environments: an artificial cilia approach†

**DOI:** 10.1039/d5lc00186b

**Published:** 2025-05-01

**Authors:** Tongsheng Wang, Ishu Aggarwal, Erik Steur, Tess Homan, Patrick R. Onck, Jaap M. J. den Toonder, Ye Wang

**Affiliations:** a Department of Mechanical Engineering, Eindhoven University of Technology 5600 MB Eindhoven The Netherlands y.wang2@tue.nl; b Institute for Complex Molecular Systems, Eindhoven University of Technology 5600 MB Eindhoven The Netherlands; c Zernike Institute for Advanced Materials, University of Groningen 9747 AG Groningen The Netherlands

## Abstract

Mixing in stagnant microfluidic environments is essential for many applications but is inherently challenging to achieve, due to the difficulty in generating effective flow disturbances at the sub-millimetre scale. Traditional passive micromixers require externally driven flow to work, while active mixers often have restrictive use cases with undesirable side-effects. In this study, we designed a 6 × 6 array of multi-directionally inclined magnetic artificial cilia that can be manufactured using a newly developed micromolding process. The array can generate strong local mixing by creating overlapping vortical flows generated by the cilia's tilted conical motion, as well as efficient global mixing by producing a complex flow pattern composed of a collection of predesigned, criss-crossing net flow vectors. Experimental results show effective mixing in stagnant microfluidic environments within 25 seconds when the cilia are actuated by a rotating global magnetic field at a frequency of 20 Hz. We performed fully coupled simulations to design the 3D motion of the cilia. Simulations of double-cilia systems with prescribed motion provide insights for designing the large array, and a simulation of the full array was performed to analyse the mixing effect in 3D.

## Abstract 2

An artificial cilia array achieves fast, efficient mixing in stagnant microfluidics within 25 seconds using crisscrossing vortices.

## Graphical abstract

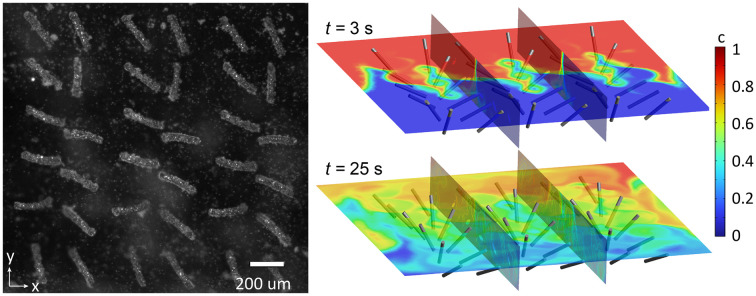

## Introduction

Mixing in a stagnant microfluidic environment is critical for many applications and yet it remains a formidable challenge.^1,2^ In a typical application scenario, different components need to be quickly and effectively mixed in a small chamber, where the fluid is not being transported in and out of the domain. The small length scales of these systems dictate that at low Reynolds numbers, mixing is governed primarily by diffusion unless an external mechanism, such as artificial cilia, introduces localized chaotic advection. This makes achieving rapid and homogeneous mixing of reactants a significant challenge for time-sensitive applications, particularly in fields such as medical diagnostics,^3,4^ drug development,^5^ and chemical analysis.^6,7^

To address these challenges, a variety of microfluidic mixing techniques have been developed, which can be broadly classified into passive and active approaches.^1,2,8–10^ Passive mixers can accelerate molecular diffusion by increasing the interface area between different fluid layers, reducing layer thicknesses or prolonging interaction time,^11,12^ through implementing static geometrical features in the microfluidic channels. Examples of passive strategies include using serpentine or zigzag microchannels,^13^ discrete through-holes,^14^ herringbone patterns,^15^ and more complex 3D structures.^4,16–18^ There are also passive channel structures that can induce vortices^16,19–21^ but these typically require higher flow rates. Although they are effective in achieving mixing, passive methods require complex and elongated channel geometries and an externally driven flow, making them unsuitable for mixing in stagnant fluids.

In contrast to passive mixers, active mixers make use of external energy, such as electric,^22–26^ magnetic,^27–29^ or acoustic^30–32^ energy to induce fluid motion for mixing. These methods can be very effective in low-Reynolds-number flow as well as in stagnant environments. Examples of active mixers include magnetic micro stirrers, acoustic mixers that use standing waves to generate flow and particle motion, electrokinetic mixers that apply electric fields to induce flow,^9^ and flow pulsation devices.^33^ However, despite their efficiency, active mixers often have restrictive use-cases and are complex in their design, fabrication, and control.^8^ For instance, magnetic bead-based micro-stirrers are difficult to control due to clustering,^4,29,34^ acoustic mixers can raise the temperature and create cavitation^2,31^ which may damage biological materials, and electrostatic mixers need an electrical field, which is undesirable for biological applications as well. In addition, active micromixers often require complex fabrication processes, and many principles (except magnetic) require tethering to an external controller, further increasing the complexity for their integration with existing workflows.

In recent years, nature-inspired artificial cilia technologies have been developed for microfluidic applications, including fluid transportation,^35–39^ particle manipulation^40^ and mixing.^9,41–44^ One of the earliest studies^9^ demonstrated the effectiveness of cilia-induced mixing in a Y-shape microfluidic device using electrostatic actuation, and various magnetic artificial cilia were developed later that are more compatible with biological materials and can be used to enhance mixing.^44–47^ Pneumatically driven millimetre-scale artificial cilia were proposed^48–50^ that are able to move in a metachronal wave and generate mixing, but it is difficult to scale down the fabrication of these cilia for microfluidic applications. In an earlier study, using an in-house developed micromolding technique, we have shown that a 1D metachronal wave generated by artificial cilia can effectively transport fluid in a microfluidic channel, without any other flow-generating asymmetry effect,^35^ and we have hypothesized that 2D metachronal motion could be used to effectively generate mixing. The new fabrication method introduced in that earlier study allows us to pre-program phase differences between cilia in the metachronal motion to optimize flow generation, and the same principle may be applied to optimize mixing. The unprecedented degree of freedom in designing flow patterns at the micrometre scale by programmed motion of individual cilia could potentially improve mixing efficiency beyond the current state-of-the-art.

In this work, we have developed a 6 × 6 array of inclined magnetic artificial cilia, each pointing at a different predesigned orientation. When actuated with a rotating magnetic field, the cilia individually perform tilted conical motion in different directions, while they collectively move in a way that resembles a 2D metachronal wave. The cilia motion was designed to enhance both local mixing by generating overlapping local vortical flows, and optimize global mixing by creating a collection of criss-crossing net flow vectors using the non-reciprocal nature of the tilted conical motion. For optimization of the cilia design, their motion was first investigated using a fully coupled simulation model, and mixing analyses were performed on small units consisting of double-cilia pairs that are pointing in different directions and moving with specific phase differences. Based on the insights generated by the double-cilia analyses, 6 × 6 cilia arrays were designed and fabricated at micrometre-scale using a micromolding technique, which was modified from our previous study^35^ for creating programmable metachronal motion. The magnetic artificial cilia array was actuated in a rotating uniform magnetic field generated by a Halbach array of permanent magnets. The mixing effects were experimentally quantified in 2D by analysing tracer particle distributions over time, with well-controlled initial conditions. 3D mixing analyses were performed using numerical simulations based on prescribed cilia motion. Our results demonstrate that the designed magnetic artificial cilia array effectively achieved global mixing within 25 seconds, driven by the combination of strong local mixing and distributive global transport. Numerical simulations confirmed that local transport vectors induced by the cilia facilitated rapid homogenization of initially separated fluids, forming an interdigitated finger pattern within just a few seconds. In contrast, the reference array, with uniformly oriented cilia, was less effective, primarily generating net fluid transport rather than creating larger interfaces for enhanced mixing.

## Results

### Fabrication of metachronal cilia arrays

**Fig. 1:**
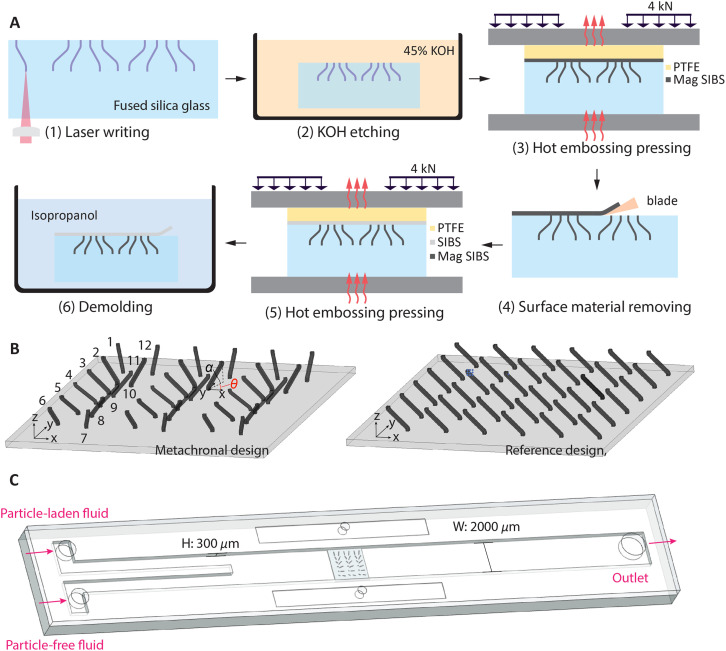
Fabrication of cilia arrays for mixing and the experimental chip. (A) Cilia fabrication process: (1) local material modification within a fused silica slide by direct writing using a femtosecond laser (FEMTOprint f200); (2) etching in 45 wt% potassium hydroxide solution bath at 85 °C with ultrasonication to obtain a mold; (3) transfer molding with a layer of magnetic particle doped poly(styrene-*block*-isobutylene-*block*-styrene), or magnetic SIBS, at 130 °C; (4) surface material removal using a blade. (5) Molding at 130 °C with a layer of pure SIBS as transparent substrate; (6) demolding of the cilia patch in isopropanol. (B) Two designs of the cilia array for mixing experiments. The cilia have a length of 240 μm, a square cross section with a side length of 24 μm, and an inclination angle *α* (the angle between cilia and the *x*–*y* plane) of 45°. The orientation angle *θ* (the angle between the projection of cilia on the *x*–*y* plane and the *x*-axis) changes in steps of 30° from cilia 1 to 12 and repeats 3 times in the metachronal design, while the reference design has the same *θ* of 90° for all cilia. (C) Assembled chip containing two inlets and one outlet. The height of the channel is 300 μm and the width is 2 mm. The cilia array is placed in the centre of the channel. This chip is designed for filling the stagnant fluid at the cilia patch location with half particle-laden and half pure fluids, and the inlets and outlets are immediately sealed after filling. The magnetic actuation and imaging setups have been reported in an earlier study^35^ and are not shown here.

In this study, we employ a modified method based on a previously developed cilia fabrication process.^35^ As illustrated in Fig. 1A (1–6), the process begins with laser machining using a focused femtosecond laser (FEMTOprint f200) to write a 3-D pattern in a fused silica glass slide. The laser beam is focused with a Thorlabs 20× objective, yielding an ellipsoidal voxel about 3 μm wide in the *x*–*y* plane and 24 μm long along *z*. The 1030 nm wavelength laser operates at 1000 kHz and 230 nJ per pulse, giving a resolution of ∼1 μm, while the writing speed is 950 mm min^−1^. Cilium dimensions are also influenced by the programmed tool path: with the 20× objective the minimum resolution in the *x*–*y* plane is 3 μm (diameter for straight cilia) and 24 μm in the *z* direction (which influences the inclined-root cilia); with a 10× objective the corresponding limits are 4 μm and 42 μm. Larger structures can be produced by proportionally enlarging the tool path without changing the optical settings.

After etching the written pattern and surface treatment, micromolds with pre-programmed structures were obtained. The detailed fabrication process for these steps shown in Fig. 1A (1–3) is available in our previous work.^35^ The molding and demolding steps are depicted in Fig. 1A (3–6). Different from the previous fabrication process, we first utilize hot-embossing to press a precursor layer of magnetic particle-doped poly(styrene-*block*-isobutylene-*block*-styrene) (magnetic SIBS) into the mold, followed by the removal of excess surface material with a blade. Subsequently, a layer of pure SIBS was hot-embossed to form a transparent substrate, which is needed for the characterization of cilia motion and mixing analysis using microscopy.

Using this fabrication method, we can create cilia arrays with pre-designed parameters, allowing for the generation of complex collective motion. In our previous work, we demonstrated this capability with partially magnetized flap-shaped cilia performing 2D motions.^35^ In this study, we extend this approach to create programmable 3D motion with fully magnetized rod-shaped cilia. Fig. 1B shows the metachronal and reference designs, where all cilia share the same dimensions: a total length of 240 μm, a square cross-section with a side length of 24 μm, a inclined angle *α* of 45°. The key difference lies in the orientation angle *θ*, the angle between the projection of cilia on the *x*–*y* plane and the *x*-axis: for the metachronal design, *θ* changes incrementally by 30° from cilia 1 to 12, while in the reference design, *θ* is fixed at 90° for all cilia. The tip-to-tip pitches for both arrays in both *x* and *y* directions are 300 μm. In the metachronal array, *θ* determines the magnetic torque experienced by different cilia at any given time, and the differences in *θ* cause phase difference in the motion of neighbouring cilia, whereas in the reference array, the cilia exhibit synchronized motion. These two designs were used to compare the mixing performance of metachronal and synchronized motion. While the cilia array is positioned in the *x*–*y* plane, a uniform magnetic field rotates around the *z*-axis. This magnetic field is generated by a motor-driven Halbach array of permanent magnets.^35^

During preliminary trials we compared several layouts that varied both cilia spacing and metachronal phase shift. Among the patterns we tested, the 6 × 6 metachronal design presented in this paper produced the shortest mixing time, so we adopted it for the main experiments. Representative video from the alternative designs have been added to the ESI† (SM movies 12 and 13†). We do not claim, however, that the chosen geometry is universally optimal. The present work aims primarily at introducing a new concept using artificial cilia that enables efficient mixing in stagnant liquids; the ideal configuration will depend on the specific channel dimensions and operating constraints of each application. To optimize mixing for specific applications, systematic studies on cilia configurations might also be needed.

Fig. 1C shows the microfluidic chip used for the mixing experiment. The chip was designed to create a stable and reproducible initial state at the location of the cilia patch, by having two inlets at one end and a single outlet at the other end, and by filling the channel through the inlets with a particle-laden fluid and a pure fluid, respectively. This approach results in the two types of fluids each occupying half of the area at the cilia region. The channel has a height of 300 μm and a width of 2000 μm. In order to achieving a reproducible initial state for stagnant microfluidic mixing, we opted to use particles for visualizing the mixing process instead of dyes, to limit the effect of diffusion. To introduce the fluids, we used a Harvard Apparatus PHD 2000 infusion syringe pump and set both inlets at the same flow rate. Once the flow stabilized, the pump was turned off, and all ports were sealed with adhesive to establish a closed environment with a stable initial state.

In the context of this paper, a stagnant environment is one in which no externally imposed flow is present, other than the flow induced by cilia. Practically speaking, an externally plumped through-flow is not present, and the flow in the domain only of the small, recirculating currents that the cilia themselves generate.

### Individual cilia motion

**Fig. 2:**
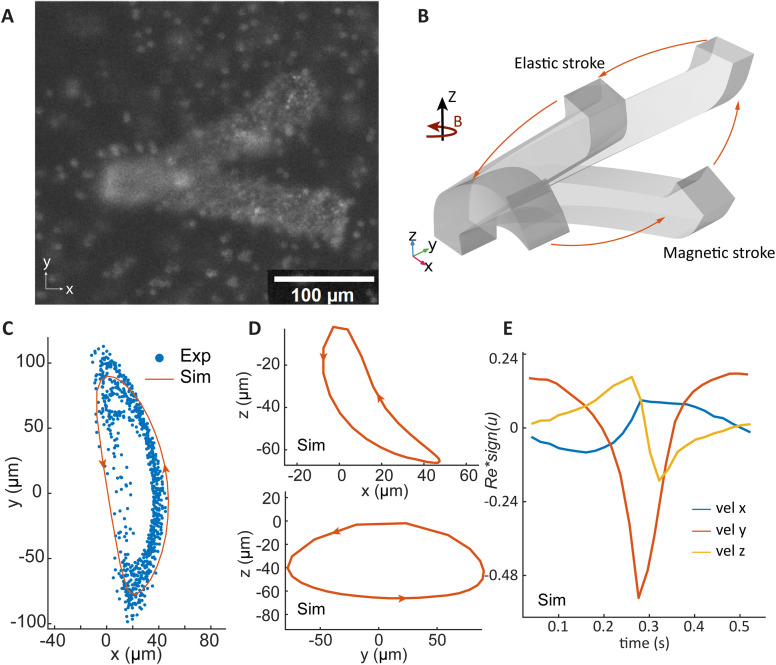
Cilia response to a rotating uniform magnetic field. (A) A top-view image showing the two extreme cilia positions under the actuation of an anticlockwise rotating uniform magnetic field around the *z*-axis. (B) COMSOL simulation result of the cilia response to the rotating magnetic field. (C) Cilia tip trajectories projected onto the *x*–*y* plane, as determined by experiment and simulation. The experimental results are collected from 12 cilia. (D) Simulation results of cilia tip trajectories projected onto the *x*–*z* plane and *y*–*z* plane, respectively. (E) Reynolds number Re calculated from cilia tip velocities in the *x*, *y*, and *z* directions determined by simulation. The absolute Re values are multiplied by the directions of the velocities.

Fig. 2A presents the top-view of a cilium at its extreme positions when actuated by an anti-clockwise rotating magnetic field at the frequency of 1 Hz. Due to the difficulty of experimentally measuring cilia motion in the *z*-direction, we conducted a fully-coupled 3D numerical simulation using COMSOL Multiphysics®, including fluid mechanics, solid mechanics, and magnetic field. Fig. 2B shows the simulation results of the cilium's motion under the same rotating magnetic field as applied in the experiments. The motion trajectory resembles a tilted conical cone and consists of two regimes: a magnetic stroke, during which the cilium follows the magnetic field, and an elastic stroke, in which the cilium sweeps back once the deflection reaches a point where the elastic torque surpasses the magnetic torque.

This motion is a combination of a 3D tilted conical motion^37,51,52^ and a 2D flap-like motion,^35,53^ and the timing of the individual motion programmed by the phase angle *θ* allows us to design the collective metachronal motion. The tilted conical motion is quite effective at generating flow, as demonstrated by both natural and artificial cilia.^54^ It is commonly induced by having the cilia follow a rotating magnetic field, which can be generated by electromagnets^28,29^ or rotating permanent magnets.^35,36^ In one cycle, a spatial non-reciprocity is created, with half of the cycle occurring close to the substrate (the so-called recovery stroke) and the other half farther away from the substrate (the so-called effective stroke). However, this principle cannot be directly applied for creating metachronal motion, since at such a small scale all cilia follow the same field direction simultaneously. On the other hand, metachronal motion has been created by using flap-like cilia with various bends at the base when they were actuated by an in-plane rotating magnetic field.^35^ The motion consists of a magnetic and an elastic stroke, with a pre-programmable timing determined by the angle of the bend at the base of the cilia. However, the motion is largely reciprocal and only through metachronal coordination at small distances can a significant flow be generated. Hence, a new design is needed to create a combinatory motion that includes both non-reciprocal single cilium motion and a metachronal collective motion in 2D that allows a non-unidirectional transport for more effective global mixing.

The tilted cilia design enables the combination of the two types of motion mentioned above. The magnetic stroke, where the cilia move closer to the substrate following the horizontally rotating field, drags along less fluid and acts as the recovery stroke. The elastic stroke, when the elastic torque stored in the cilia exceeds the magnetic torque so the cilia move back in a faster motion following a trajectory further away from the substrate, and acts as the effective stroke of the cycle. Fully coupled numerical simulation captures the details of the 3D motion as shown in Fig. 2B, quantitatively characterizing the spatial asymmetry and timing of the cilia motion, which enables the design of a more complex collective motion. The benefit of this design and actuation method is that the shape of the motion is independent of the direction of the cilia in the *x*–*y* plane, so we can now design the phase difference between cilia.

Fig. 2C shows the comparison of the experimental and simulation results of the cilia tip trajectories projected onto the *x*–*y* plane. The experimental data was collected from 12 cilia under the same magnetic field. Although the experimental results show some variation due to minor fabrication inaccuracies, the simulation results generally match with the experimental observations. Fig. 2D shows the cilia tip trajectories projected onto the *x*–*z* and *y*–*z* planes, providing clear evidence of spatial asymmetry, showing that the magnetic stroke is closer to the substrate. Since the experiment can only obtain *x*–*y* projections, we only show simulation data here. Fig. 2E presents the Reynolds numbers (Re) calculated from the velocity components in the *x*, *y*, and *z* directions obtained from simulation, using the cilia length of 240 μm as the characteristic length. To aid in interpretation, the Re is multiplied by the sign of the cilia tip velocity in each direction. The maximum Re values are similar in the *x* and *z* directions. In the *y* direction, the maximum Re during the elastic stroke is approximately twice that of the magnetic stroke. Despite the temporal asymmetry, the inertial effects are negligible due to the small Re.

### Mixing induced by double-cilia systems with phase differences

To design a cilia array with optimal mixing performance, we first analysed the mixing behaviour induced by a double-cilia system, which can be considered as the smallest functional unit for this purpose. The analysis was conducted through 3D simulations in Ansys Fluent, with the cilia motion prescribed based on experimental measurements and fully coupled 3D simulation results from COMSOL presented in Fig. 2B. This approach allowed us to reduce the computational resources required and to efficiently generate insights for the design of larger arrays. For the initial conditions, we set the concentration of one half of the domain to 1 and the other half to 0, while the diffusion coefficient was set close to zero to represent the condition of negligible diffusion in the experiments. The phase difference between the cilia was introduced by varying the orientation angle *θ* of cilium 2 while keeping *θ* the same for cilium 1. All cilia were prescribed to perform the same tilted conical motion but with different orientations determined by the initial starting angle.

**Fig. 3:**
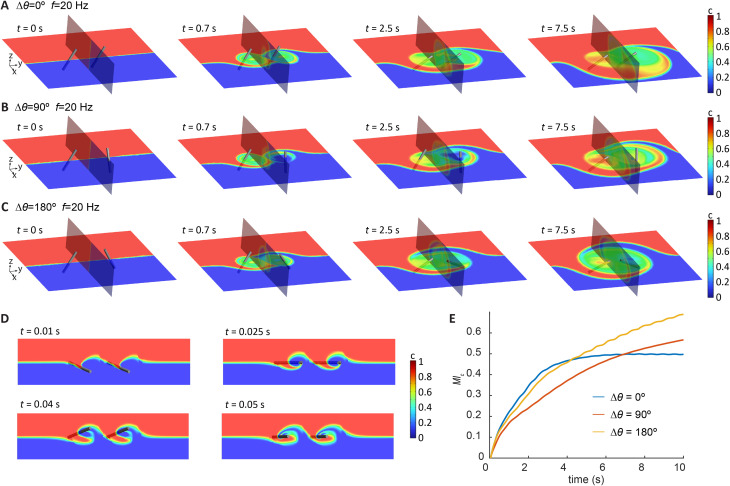
Mixing analysis of double-cilia systems with different relative orientations. (A–C) Simulation results of mixing for double-cilia systems with the *θ* (phase) differences of 0°, 90°, and 180°, respectively. In all setups, all cilia have the same initial tip-to-tip distance and have the same tilted conical motion but with different directions. In the simulation, the diffusivity is set close to 0 to show only the effect of convection. (D) The evolution of the concentration profile during the first beating cycle with the phase difference of 0°, seen from the top view. (E) The mixing index over time of the entire domain with the cilia phase differences of 0°, 90°, and 180°, respectively.

Fig. 3A–C visualizes the concentration distribution and its evolution over time for double-cilia systems with phase differences of 0°, 90°, and 180°, respectively. The cilia beating frequency is 20 Hz actuated by an anticlockwise (ACW) rotating magnetic field. The simulation domain measures 1300 μm in the *x* direction, 1200 μm in the *y* direction, and 300 μm in the *z* direction, with the tip-to-tip distance between the two cilia set at 300 μm. The colour scale represents concentration levels, with red indicating the highest concentration (*c* = 1) and blue representing the lowest concentration (*c* = 0). Each panel includes two planes: the *x*–*y* plane at *z* = 150 μm from the substrate and the *y*–*z* plane at the midpoint between the cilia tips. The time points in the figures are not equally spaced, as the concentration profile changes rapidly during the initial stages of mixing and stabilizes later, necessitating a higher frequency of snapshots in the early phase of the process.

The figures illustrate how the different configurations of neighbouring cilia affect mixing. Complex flow patterns emerge from the rather simple cilia motion, and the relative position and phase of the two cilia significantly impact the mixing performance. In all three cases, the left-side cilium 1 quickly achieves good local mixing in the early stages, while the difference in the subsequent development is determined by the phase difference. For a phase difference of 0°, both cilia generate a net flow toward the −*y* direction, which transports high-concentration fluid into the ‘active zone’ of the cilia. By the end of the process, the zone is largely filled with high-concentration fluid. For a phase difference of 90°, the right-side cilium 2 generates a net flow in the *x* direction, while the left-side cilium continues to drive flow toward the −*y* direction. The combined effect likely produces a net flow at a 45° angle between the *x* and −*y* directions. As shown in the figures, the final concentration distribution is similar to the 0° case but slightly rotated, with the high-concentration fluid primarily accumulating in the upper-left region. In the case of a 180° phase difference, the left-side cilium 1 generates a net flow in the −*y* direction, while the right-side cilium 2 generates a flow in the *y* direction. These opposing flows induce convection that promotes the exchange of fluids in a distributive way, while cancelling out the net transportation, resulting in a centrally symmetric concentration distribution and a large, well-mixed domain by the end of the process.

To quantify the effect of mixing, the mixing index (MI_c_) is calculated based on concentration distribution within the computation domain using the following equation:^55^1
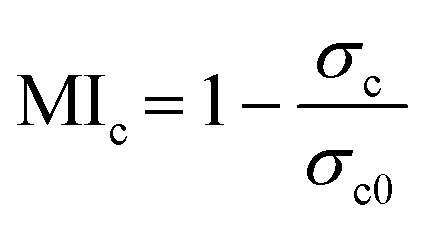
2
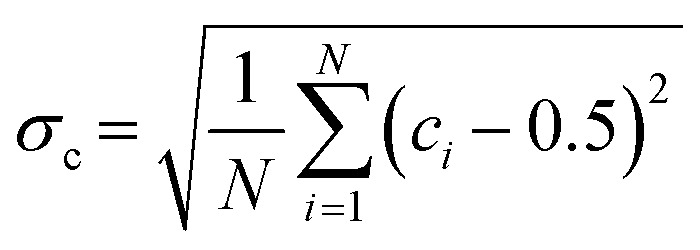
where *σ*_c_ denotes the intensity of segregation, *σ*_c0_ is *σ*_c_ at the initial time, which is 0.5, *c*_*i*_ is the concentration at point *i*, *N* is the total number of points. At each time slice, the points are equally spaced within the domain with a spacing of 5 μm in the *x, y* and *z* directions.

Fig. 3E shows the global mixing effect across the double-cilia domain. While the 0° phase difference shows strong performance initially, its effectiveness diminishes as the process continues, whereas the mixing effects for 90° and 180° phase differences continue to improve, with 180° being the best due to the reasons discussed above, namely the distributive local flows and the cancelation of the global net flow. These results suggest that for a larger array, achieving efficient global mixing requires it to generate a flow pattern with similar characteristics.

From these experiments, we can see that the tilted conical motion is quite effective at generating local mixing, but global fluid transport can significantly impact the result. Local transport flows are needed to reduce the influence of initial concentration distribution. However, global net flow might not be desirable either, as shown in Fig. 3A, since a global net flow might remove the liquid prematurely out of the active zones of the cilia and results in a reduction of total mixing efficacy. This insight led us to design an array that can create flow vectors that point at different directions resulting in zero net flow, to generate distributive transport effect to promote global mixing.

### Collective cilia motion and the induced mixing effect

**Fig. 4:**
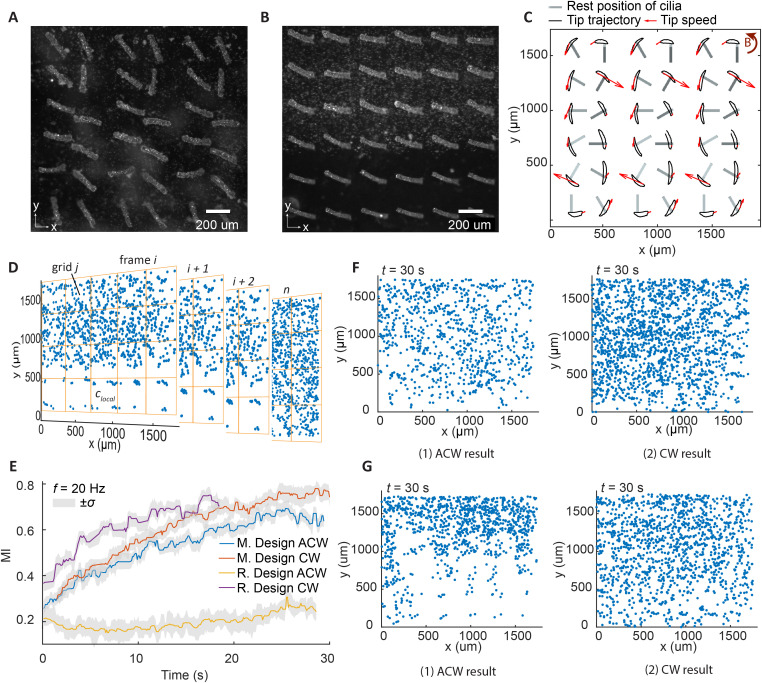
Mixing results from experiments using large cilia arrays. (A and B) Top-view image of cilia arrays of metachronal and reference designs according to the schematic diagram in Fig. 1B. (C) Experimentally measured cilia tip trajectories projected on the *x*–*y* plane of the metachronal array under the ACW actuation of 1 Hz; the rest positions of the cilia are also indicated. The phase differences between the cilia motions are shown with the red arrows, which indicate the direction and magnitude of the tip velocity of each cilium at one instant. (D) Processed images of particle distribution for the characterization of mixing at several timepoints. (E) The mixing index over time for both arrays at a cilia beating frequency of 20 Hz. Data is averaged from 3 experiments, with *σ* being the standard deviation. The seemingly superior mixing performance from the reference array with CW actuation is due to the limitation of the characterization method, as explained in detail in the text. (F and G) Mixing visualization of the metachronal design and the reference, respectively, after anticlockwise (ACW) and clockwise (CW) actuation for 30 seconds with a cilia beating frequency of 20 Hz.

Building on the insights obtained from the analysis of the double-cilia systems, we designed the 2D metachronal cilia array shown in Fig. 1B, and made another array as a reference, without varying cilia orientation as a reference, as shown in the same panel. Fig. 4A and B show top-view images of the fabricated cilia arrays for both designs. Both arrays were placed within a magnetic field, which caused the cilia in both images to orient towards the field. Fig. 4C shows the experimentally measured tip trajectories of cilia projected on the *x*–*y* plane, for the metachronal array under ACW actuation at 1 Hz. The black rods show the resting positions of the cilia. The red arrows indicate both the direction and magnitude of the tip velocities of different cilia at the same time point, showing the phase differences in the cilia motion. The tip trajectories for the reference array are not shown, as all cilia in this design perform synchronized motion.

The mixing index is quantified based on the analysis of tracer particle distribution.^41^ The original videos were recorded at a frame rate matching the same magnetic field rotation frequency, allowing us to filter the motion within a cycle. Each frame captures the particle distribution after one complete rotation of the magnetic field. Particles were detected from these videos using Fiji,^56,57^ and then the data was transferred to MATLAB for further processing to remove the images of the cilia for intensity of segregation measurements based on pixel values. Further details on the data processing steps are provided in the ESI.† The mixing index was calculated from the processed particle distribution data. As shown in Fig. 4D, the area of interest was divided into several regular and uniform grids in each frame. For each grid *j*, the local particle concentration *p*^*j*^_local_ was calculated as the ratio of the number of particles within the grid to the grid area d*A*, along with the mean particle concentration *p*^*i*^_mean_ for each frame *i*. This process was repeated for all frames, and the mixing index was ultimately calculated using the following equation:^41^3
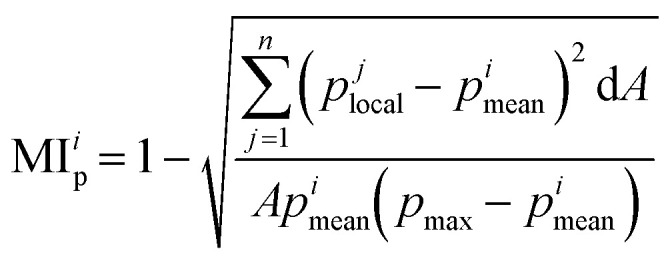
where *A* represents the total area of interest, d*A* is the area of grid and, *p*_max_ is the maximum particle concentration of all frames. Note, that the chosen grid size can influence the results, as too big or too small grids can both lead to a low mixing index. We performed some trial experiments and chose a medium grid size for a good representation. Another important thing to note is that MI_p_ derived from experimental data has a different calculation method from MI_c_ derived from simulation results, due to the difference in the types of data, which is discussed below.

Since the mixing domain was open on two sides to additional fluids, the experimental characterization method has a limitation in determining the absolute concentration as it cannot track all particles moving in and out of the domain, and the optical images can show only a portion of all particles (depth of focus is about 25 μm). Therefore, MI_p_ is determined based only on the particle distribution of the current frame, without cross referencing with frames taken at other timepoints, unlike for MI_c_ derived from numerical simulation, where the concentration profile in 3D is used and the domain has a fixed total value. This limitation brings some complications for analyses, especially when comparing the mixing effects of different arrays, as discussed below. Later, we will discuss numerical analysis results which provide details of mixing effects in 3D as additional support to the experimental characterization.

Fig. 4E shows the evolution of the mixing index (MI_p_) for the two arrays under both ACW and clockwise (CW) actuation. The grey shading represents ±1 the standard deviation range. From the figure, we can observe that the metachronal array exhibits strong mixing performance under both ACW and CW actuations, with the standard deviation areas overlapping for most of the time. The MI_p_ rapidly increases from 0.2 to approximately 0.7 within 25 seconds. This observation is further supported by the visualizations of particle distribution in Fig. 4F, where particles are evenly distributed across the field for both ACW and CW actuations (ESI† SM movies 5 and 6).

For the reference cilia array, the results under ACW and CW actuations differ significantly. At a first glance, CW actuation seems to generate even better mixing than the metachronal design, while ACW actuation generates very poor mixing. This discrepancy arises due to the aforementioned limitation of the calculation method of MI_p_, and further analysis reveal that both directions indeed generate poor mixing. Since the cilia array performs synchronized and monodirectional tilted conical motion, which generates a collective net flow along the *y* axis, they effectively drive the particle laden fluid or the pure fluid into each other's initial domain, depending on the direction of actuation, and keep on replenishing the same fluid from outside of the cilia area. As shown in Fig. 4G, under ACW actuation, regions initially filled with particles in the upper part of the field become devoid of particles. Conversely, under CW actuation, the domain gets filled with particle laden fluid over time. This indicates that the cilia array creates a global net flow towards the positive *y* axis under ACW actuation and towards the negative *y* axis under CW actuation, but there is very limited transport in the *x*-direction. If the cilia were all rotated by 90 degrees, it would effectively create a pump that drive the fluid along the channel and it can be expected that there would be very limited amount of mixing in that case as well.^36^ Therefore, the MI_p_ results of the reference array are determined mainly by a shift of the initially partitioned fluids rather than by true mixing.

We also measured the mixing performance of the metachronal cilia array under an external flow of 25 μL min^−1^ to compare with mixing in stagnant fluid, as shown in the ESI† (SM movie S11). The results indicate no significant mixing effect under this flow condition. This is because that the externally driven flow at this flow rate replaces the fluid in the entire cilia region in a matter of 3 seconds, causing the mixing effect to be overwhelmed by the incoming flow. Hence this method is not directly suitable for mixing in a flow condition. The metachronal cilia array reported here was conceived expressly to close the gap in rapid mixing techniques for stagnant fluids. Under a continuous background flow the externally generated advection convects the cilium-induced vortices downstream before they can interpenetrate, so the net mixing effect becomes marginal, which is an inherent limitation of this actuation mode in dynamic microfluidic environments. For such situations we have already introduced an alternative design, the integrated magnetic microwalls described in our earlier work,^41^ which can induce effective mixing under continuous background flow conditions. Systematically tuning magnetic-field amplitude and frequency to achieve robust performance in both stagnant and flowing regimes is an important objective, but this lies beyond the scope of the present study and will be pursued in future investigations.

Since the designed metachronal array does not generate a global net flow, but only spreads the local mixing throughout the area *via* local net flows in different directions, one can argue that this design can generate good mixing independent of the initial condition with regard to the distribution of the target substance. This is particularly important for mixing in stagnant fluids, where the initial distribution is usually unpredictable, unlike in a laminar flow condition, and hence a mixer that can be effective regardless of the starting situation is highly desirable.

### Analysis of mixing in 3D by numerical simulation

**Fig. 5:**
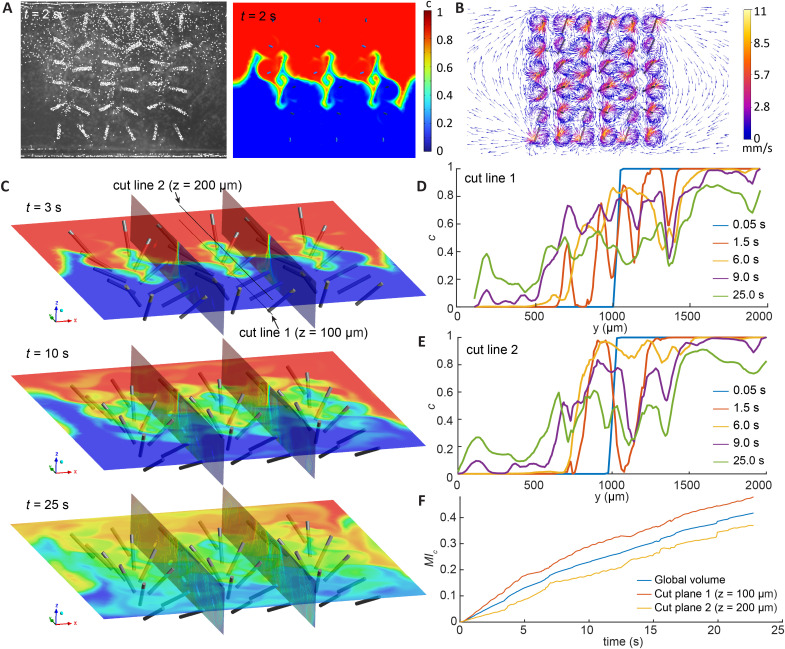
Analysis of the flow field and the mixing performance in 3D using numerical simulation. (A) Comparison of particle distribution determined by experiment and the concentration distribution determined by simulation at 2 s, showing an interdigitated finger pattern. The complete videos can be found in the ESI.† (B) Velocity vectors in the plane *z* = 150 μm at 2 s, showing the shape and magnitude of the local vortices. The video of a full cycle can also be found in the ESI.† (C) Mixing result shown in 3D using several cutplanes at different time points. (D and E) The concentration distribution of cutlines 1 and 2 at different time points, respectively. Note that at 1.5 s, oscillatory profiles emerge, showing the effect of local transport vectors in ‘dosing’ the different fluids into each other's domains. (F) The global mixing index as well as the mixing index in two cutplanes at different *z* levels calculated from the concentration distribution over time.

Since the experimental setup only allows for top-view measurements of the mixing effect, which cannot resolve the particle distribution in the *z* direction, we performed 3D simulations to investigate mixing in the entire volume. Fig. 5A compares the experimental and simulation results with both beating frequencies set at 20 Hz, showing that the concentration distribution in the simulation matches the particle distribution observed in the experiment, and the results show an emerging interdigitated finger pattern that increases the interfacial area between the fluids. The complete evolution of the mixing process is available in the SM movie S10.†Fig. 5B shows the fluid velocity vectors at *t* = 2 s, highlighting the local vortices generated by the cilia and the overall complex global flow field. These local vortices contribute to local mixing, while the net flows produced by cilia with varying orientations spread the mixing effect globally.

The current experimental setup takes images in the *x*–*y* plane with a large depth of focus in the *z* direction, so direct validation of the mixing efficiency in different planes along the *z* axis is not feasible. We therefore performed numerical simulations to help investigate the complete velocity field and the 3d mixing effects. The computed mixing time and overall trend agreed well with the experimental mixing index. To further cross-check the model, we compared characteristic flow structures at the onset of mixing. Fig. 5A contrasts the finger-like pattern obtained from the experiment with that predicted by the simulation, and the two images display the same spatial features. The combination of experimental and simulation results provides a comprehensive image of the mixing performance of the cilia array.

Fig. 5C depicts the evolution of the concentration distribution in 3D induced by mixing using the metachronal cilia array. In the early stages, the concentration distribution forms distinct patterns, which become increasingly complex and less recognizable as mixing progresses. Despite this apparent complex behaviour, the flow within the domain remains laminar, as the maximum Reynolds number (Re) reaches only around 2 at a cilia beating frequency of 20 Hz—far below the typical threshold for turbulence (Re ≈ 2000). Nevertheless, the convection induced by arranging the cilia with different orientations has led to turbulence-like evolution of the concentration distribution.

Fig. 5D and E illustrate the concentration evolution over time along cutline 1 and 2, which are located at *z* = 100 μm and 200 μm respectively in the middle of the cilia array along the *y*-axis, as defined in Fig. 5C. Initially, a step-like distribution is observed for both cutlines, with a clear separation at *y* = 1000 μm, indicating an initially sharp concentration gradient. After 1.5 seconds, we see an oscillatory concentration distribution, with the bottom cutline having a smaller ‘wavelength’. These oscillations become less pronounced as time advances, resulting in quick smoothening of the concentration gradient. Apart from some differences at the beginning, the two cutlines show similar evolution in the profiles, indicating similar performance in mixing throughout the height of the chamber. Note, that the profiles at 1.5 s suggest that the multidirectional tilted-conical motion can indeed ‘dose’ different fluids into the domains of consecutive cilia, leading to quick homogenization afterwards thanks to the strong local mixing effect of the cilia.

Fig. 5F shows the mixing index calculated for the entire domain using eqn (1), as well as at two cutplanes at *z* = 100 μm and 200 μm. They show similar trends as the experimental results shown in Fig. 4E, indicating that for the current experimental setup, where the height of the chamber is significantly smaller than the width and length, the 2D experimental characterization method can already provide good accuracy for analysing mixing.

## Conclusions

In this work, we designed and fabricated a 6 × 6 magnetic artificial cilia array using a modified femtosecond laser machining process for mixing in a stagnant microfluidic environment. When actuated with a rotating Halbach array that generates a uniform rotating magnetic field, the newly designed cilia all exhibit a tilted conical motion, but with directional and phase differences between neighbours, and they form a collective motion that resembles a 2-dimensional metachronal wave.

The design of the array, optimized for mixing, was based on the insights obtained from numerical analyses of double-cilia systems. On one hand, these numerical simulations results showed that while an individual cilium can induce rapid local mixing in a small ‘active zone’, achieving global mixing requires homogenization over the entire fluid domain by generating local net flows that promote exchange of fluid in different directions. On the other hand, the results suggested that a global net flow could indeed adversely impact mixing due to unidirectional transport and insufficient local exchange of fluid, reducing the effectiveness of mixing in the overall domain. These insights led us to the design of the cilia array with a collection of transport vectors of varying orientations to facilitate the transition from local to global mixing. To make an analogy, a single cilium functions like a localized mixing factory, where raw materials enter, and mixed fluids are transported out. The local net flows, moving in different directions, act as the transport mechanism for these materials. When combined, these flows create a dynamic fluid field in which the fluids spread out in all directions, efficiently achieving global mixing.

Experimental results demonstrated that the designed cilia array achieved effective mixing within 25 seconds at a beating frequency of 20 Hz. Numerical simulation confirmed the mixing effect in 3D and provided details over the time evolution of the concentration profiles at different height levels. The results show that two initially separated fluid components quickly form an interdigitated finger pattern after just a few seconds, and a complex pattern emerges afterwards,^4,17,18^ leading to global mixing in a short amount of time. Time-dependent concentration profiles of cutlines at different positions show the effect of local transport vectors in ‘dosing’ fluids that were initially partitioned into each other's domains, and a quick homogenization afterwards thanks to the strong local mixing induced by cilia rotation. A reference cilia array, with all the inclined cilia pointing at the same direction, was shown to create a net transportation that shifts the fluid partitions rather than creating larger interfaces, and therefore is less effective in mixing than the designed array.

Our metachronal magnetic cilia array can induce mixing in stagnant fluids within 25 s under a 0.22 T magnetic field rotating at 10 Hz. Acoustic mixers can achieve mixing on sub-second—and even millisecond—timescales, but they require bonded piezo-transducers and RF electronics. These components draw milliwatt- to watt-level power that can induce heating, and requires complicates assembly and packaging.^28,58^ Electro-kinetic devices are shown to achieve mixing in 0.1 s. They rely on patterned micro-electrodes and high voltages that can induce heating, electrolysis or other undesirable effects on biological materials.^14^ Thermal-convection mixers are more straightforward to fabricate and can operate in larger volumes, but the intrinsic mechanism can damage temperature-sensitive samples or affect the analysis.^59^ Our cilia, by contrast, can be reliably produced in a molding process, which can be readily scaled. They are actuated in an untethered way and generate mechanically induced flow without significantly heating the fluid, and they are especially suited for handling biological materials in microfluidic environments. For other less sensitive applications, one might consider combining different mixing techniques, such as a magnetic–acoustic hybrid mixer, which could potentially shorten the mixing time further. Indeed, exploring acoustic effects for fast local mixing combined with cilia driven convection can be an interesting direction for future work.

On one hand, this work has introduced a new approach to design complex flow for mixing in stagnant fluids using magnetic artificial cilia. The fabrication technique allows a large degree of freedom in design, which we have not fully exploited in the current study. Hence, it is perceivable that the design can be further optimized based on the principle of combining local mixing and distributive global transport. For example, other modes of cilia motion can be incorporated by employing complex geometries^60,61^ and inhomogeneous magnetic properties^53^ and combinations of these different types of motion can possibly lead to even faster local mixing, and/or more effective homogenization over the entire domain. Moreover, different geometries of the chamber and nature of the fluids to be mixed may require different design of the cilia, the array, and the actuation method, and to design ‘for purpose’ calls for more advanced design tools that include a more efficient simulation framework and better characterization methods.

On the other hand, the micromolding technique has been proven to be quite versatile for making different actuators for various microfluidic applications, including fluid pumping and on-demand mixing in a flow channel, and it has the advantage over other fabrication methods in the possibility of scaling up for industrial applications. It can be foreseen that other types of microtransducers can be made using this technique for applications besides fluid manipulation, and together they can form a multifunctional ‘toolbox’ to offer unique functionalities in biological analysis, medical, and engineering applications.

## Materials & methods

### Fabrication process of the cilia array

The femtosecond laser system FEMTOprint f200 aHead^35^ was used to fabricate the cilia mold and the chip. The cilia have a square cross-section, as this shape is more convenient for the opal shaped laser voxel to write. The laser system operates by moving in the *x*–*y* plane and in the *z* direction, and the laser focuses on an effective voxel with an ellipsoidal shape, featuring a 3 μm short axis in the *x*–*y* plane and a 24 μm long axis in the *z* direction. Due to this configuration, fabricating cilia with a circular cross-section, particularly at the inclined root, would be difficult and require complex control parameters. The laser path was designed using AlphaCAM with a tool setting tailored to the laser parameters. Detailed physical parameters of the laser, along with the subsequent etching and surface treatment, are provided in our previous paper.^35^

Since the camera captures images from the top-view perspective, a transparent substrate is required. To achieve this, we first press the magnetic SIBS, containing 70 wt% superparamagnetic carbonyl-iron powder (purchased from Sigma-Aldrich), into the mold using Specac hydraulic press with a heating unit. The temperature is then raised to 180 °C, and the mold filled with magnetic SIBS is heated for 5 minutes. Once the magnetic SIBS softens, a blade is used to remove the surface layer of magnetic SIBS. Subsequently, a layer of pure SIBS is pressed on top with the same temperature and duration to form the transparent substrate. The pressure was then released, and the cilia patch was manually demolded with the mold submerged in isopropanol.

A single glass mold fabricated with our protocol can be reused over many embossing cycles without measurable degradation. In the present study the same mold continued to produce defect-free cilia after more than one year of intermittent use, which confirms the robustness of the process. Although we chose a 6 × 6 array for clarity in characterization, the array size can be readily increased. Hot embossing transfers all features in one step, so the array size is limited only by the size of the mold and the press. Larger mixers can therefore be obtained by repeating the 6 × 6 unit or by writing a larger pattern directly into the glass mold.

The transparent, non-magnetic SIBS substrate layer was introduced solely to facilitate optical access during experiments. In applications where *in situ* observation is unnecessary, the cilia can remain attached to the original embossed substrate, which further simplifies production. The Halbach array and actuation stage used in our laboratory were built to generate and control a rotating uniform magnetic field for fundamental studies. Other simpler actuators, such as rotating magnets, might also be used, as long as similar motion of the cilia array can be reproduced (which might require some changes in the cilia geometry such as the tilting angles). Therefore, the fabrication and operation of these cilia array mixers can be scaled.

In the present study each cilia array was driven for more than fifty thousand cycles, and we did not observe any sign of mechanical or magnetic degradation. This finding agrees with our earlier work on flap-shaped cilia, where the structures remained fully functional after well over one hundred thousand cycles. Mixing experiments were repeated many times by re-establishing the initial concentration field and actuating the same array. We also repeated the entire protocol one year after the first trials, using the original mold, and obtained the same mixing indices and mixing time.

### Visualization of the mixing effect

To obtain natural buoyancy of the particles, we first prepared the particle-free fluid using deionized (DI) water and sodium chloride to match the density of the fluid to that of the particles. After preparing the particle-free fluid, we divided it and added particles to one portion to create the particle-laden fluid. The particles used were 5 μm carboxyl-functionalized polystyrene particles, sourced from microParticles GmbH. The particle-laden fluid was sonicated for 15 minutes to ensure uniform dispersion.

During the filling process, we used a Harvard Apparatus PHD 2000 infusion syringe pump to inject the fluids. Once the flow stabilized and distinct layers of particle-laden and particle-free fluids were formed, the pump was stopped and all inlets and outlets were sealed, establishing a steady initial state with two equal fluid partitions. The initial state can be viewed in the SM videos S5–S7.† Following this, the actuation was initiated and images were captured at a frame rate synchronized with the actuation frequency. The recorded videos were analysed using Fiji software with the TrackMate plugin.^56,57^ TrackMate detects particles and traces their trajectories; however, in our case, we only used it to detect particle positions. Tracking trajectories was challenging because the particles performed 3D motion, frequently moving in and out of the focal plane, so complete trajectories cannot be obtained. The detection data were then transferred to MATLAB for further processing and analysis.

### Quantification of MI based on particle distribution

In the captured images, the particles in the fluid and the magnetic powders inside the cilia appear similar, making it difficult for detection algorithms to distinguish between them. To address this, we developed a custom filtering algorithm in MATLAB to remove the magnetic powders from the data. Since the camera captures images at a frame rate synchronized with the actuation frequency, the position of the cilia remains unchanged across frames. By comparing the detected particle positions across three frames, we filtered out particles that appeared in the same location in all three frames, as these were likely to be the magnetic powders within cilia and tracer particles adhering to the cilia.

The mixing index (MI_p_) calculation algorithm was applied after the data was processed. Each frame was divided into small grids, where the local particle concentration *p*^*j*^_local_ was computed. After several trials, a grid size of 260 μm was chosen for optimal performance. If the grid size was too small, the MI_p_ evolution become unstable, and if the grid size was too large, it is not possible to adequately capture the evolution. Afterwards, the mean particle concentration *p*^*i*^_mean_ for each frame and the maximum particle concentration *p*_max_ were calculated. Finally, the MI_p_ was computed using eqn (3).

When an external flow was imposed, the cilium-generated vortices were affected and transported downstream, so actuating the array did not achieve mixing. This outcome confirms that the present architecture is only suitable for stagnant liquids, while our previously reported magnetic microwalls^41^ can be used for mixing under continuous flow. We have not yet explored the effect of pH or ionic strength. The cilia are made from styrene–isobutylene–styrene (SIBS), a biostable elastomer that retains its mechanical properties across a wide pH range and has passed rigorous long-term tests for implant applications.^62^ No degradation of SIBS has been reported in living systems to date, and we therefore expect the mixer to remain operational in aqueous environments.

### COMSOL and ANSYS Fluent 3D simulation model

We developed a 3D fully-coupled COMSOL® simulation model to analyse the cilia's 3D motion. The governing equations and material parameters are consistent with those used in our previous 2D model.^35^ However, in this 3D model, we implemented a segregated solver to improve computational efficiency. The magnetic field, solid mechanics, and moving mesh components were solved using the PARDISO solver, while fluid mechanics were handled by the GMRES solver. The time discretization was performed using the implicit backward differentiation formula with a maximum order of 5.

For the subsequent simulations of the double-cilia system and the cilia array, we prescribed the cilia motion based on the validated fully-coupled simulation to reduce computational time. These simulations were performed using FLUENT, which offers significantly faster simulation speeds compared to COMSOL, owing to its use of the finite volume method. To simulate the mixing effect, we employed the species transport model in FLUENT. In the initial state, we set the concentration to 0 in the upper layer of the channel and 1 in the lower layer. The diffusion coefficient was set to 1 × 10^−20^ m^2^ s^−1^ to ensure a environment of negligible diffusion while avoiding calculation errors that can occur when the coefficient is set to 0. The pressure-inlet and pressure-outlet boundary conditions are applied at the left and right boundary, while other are no-slip walls. The convection–diffusion equations used are as follows:

4 ∇·**J**_*i*_ + **u**·∇*c*_*i*_ = 0

5 **J**_*i*_ = −*D*_*i*_∇*c*_*i*_

where ***J***_*i*_ is the molar flux (mol m^−2^ s^−1^), *D*_*i*_ is the diffusion coefficient (m^2^ s^−1^), *c*_*i*_ is the concentration (mol m^−3^), **u** is the background flow field. In our case, *D*_*i*_ is close to 0, so the equations can be simplified as:

6 **u**·∇*c*_*i*_ = 0

where the concentration evolution is determined solely by the background flow field induced by the cilia. While particle tracing could be another option for simulating the mixing effect, the interaction between particles and the cilia walls often leads to computational failures. Taking these factors into account, we opted to use the species transport model to simulate the mixing effect.

In the present Fluent model, the cilia trajectories are prescribed rather than solved in a fully coupled manner. The analysis in the SM video 4† shows that the magnetic driving force acting on each cilium is more than three orders of magnitude larger than the corresponding fluid force, and the motion is therefore dominated by the magnetic force. Because of that, we neglected the flow-induced interactions between adjacent cilia in the simulation.

## Author contributions

Conceptualization: TW, JdT, YW. Experiments: TW. Simulations: TW, IA, PRO. Collecting data, coding and analysing: TW. Writing and editing: TW, YW, JdT, ES, TH, IA, PRO. Supervision and financial support: JdT, YW, ES, TH, PRO.

## Conflicts of interest

Authors declare that they have no competing interests.

## Supplementary Material

LC-025-D5LC00186B-s001

LC-025-D5LC00186B-s002

LC-025-D5LC00186B-s003

LC-025-D5LC00186B-s004

LC-025-D5LC00186B-s005

LC-025-D5LC00186B-s006

LC-025-D5LC00186B-s007

LC-025-D5LC00186B-s008

LC-025-D5LC00186B-s009

LC-025-D5LC00186B-s010

LC-025-D5LC00186B-s011

LC-025-D5LC00186B-s012

LC-025-D5LC00186B-s013

LC-025-D5LC00186B-s014

## Data Availability

The data supporting this article have been included as part of the ESI.†
