# Supplementary material for: Designing enhanced mixing in stagnant microfluidic environments: an artificial cilia approach
Source: Lab Chip. 2025 May 1;25(16):3979–92. doi: 10.1039/d5lc00186b (PMC12076189; doi:10.1039/d5lc00186b)
Supplement: LC-025-D5LC00186B-s006 [file LC-025-D5LC00186B-s006.pdf]

Supplementary Information (SI) for Lab on a Chip.
This journal is © The Royal Society of Chemistry 2025

Supplementary Materials for

Designing enhanced mixing in stagnant microfluidic environments: an artificial cilia approach

Tongsheng Wang *et al.*

*Ye Wang. Email: y.wang2@tue.nl

This PDF file includes:

Supplementary Text
Figs. S1 to S4

Other Supplementary Materials for this manuscript include the following:
Movies S1 to S13

Supplementary Text

SM 1: Magnetic actuator

Fig. S1 shows the experiment setup for magnetic actuation and data collection. A magnetic actuator was constructed to drive the cilia at high frequencies. To ensure a strong and stable magnetic field across all frequencies, a Halbach array of permanent magnets was used instead of an electromagnet. The array consists of 16 neodymium magnets (10×10×40 mm), producing a uniform field of 0.22 Tesla around the center. A DC motor with an encoder drives the array via a timing belt, allowing light to pass through the optical path. The chip is positioned in the central plane of the array, with the cilia patch located at the center to ensure optimal field uniformity.

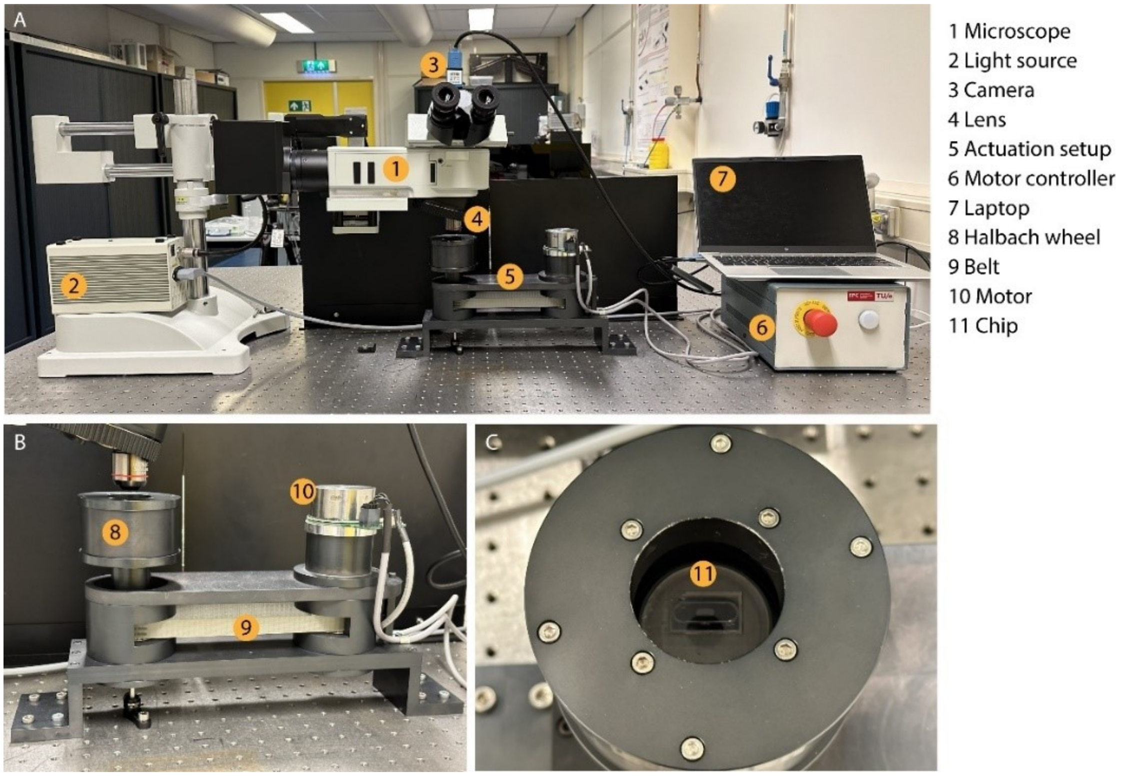

Fig. S1: Magnetic Actuator Setup. (A) The experimental setup includes a microscope, the magnetic actuator, and a computer. (B) The magnetic actuator comprises a DC motor, a Halbach wheel, and various mechanical components for the connections. (C) The chip is positioned on a stage holder at the center of the Halbach wheel.

SM 2: Filtering of fake particles

During particle detection using ImageJ, the magnetic powders within the cilia are often mistakenly detected as particles. To address this, it is necessary to filter them out. We export the detected particles to MATLAB and compare the particle positions across three frames. If a particle is present at the same location in all three frames, it is considered a false particle, since the cilia remain at the same location between frames due to the camera capturing images at a frame rate synchronized with the actuation frequency. Fig. S2 shows the particles detected by ImageJ (1), the exported particles (2), and the particle distribution after filtering.

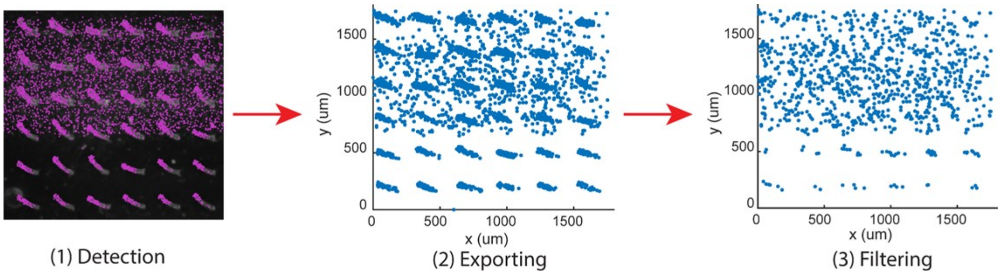

Fig. S2: Filtering process of fake particles.

SM 3: Settings of the simulation models

Fig. S3 illustrates the boundary conditions in FLUENT. The geometry of the domain and the position of the cilia for the large cilia array are consistent with the experimental setup. The left boundary is set as a pressure inlet, while the right boundary is a pressure outlet. The cilia motion is prescribed as tilted cone with opening angle 35° and tilting angle 50°. The rotation speed of the cilia is determined based on the results from the fully coupled COMSOL simulation.

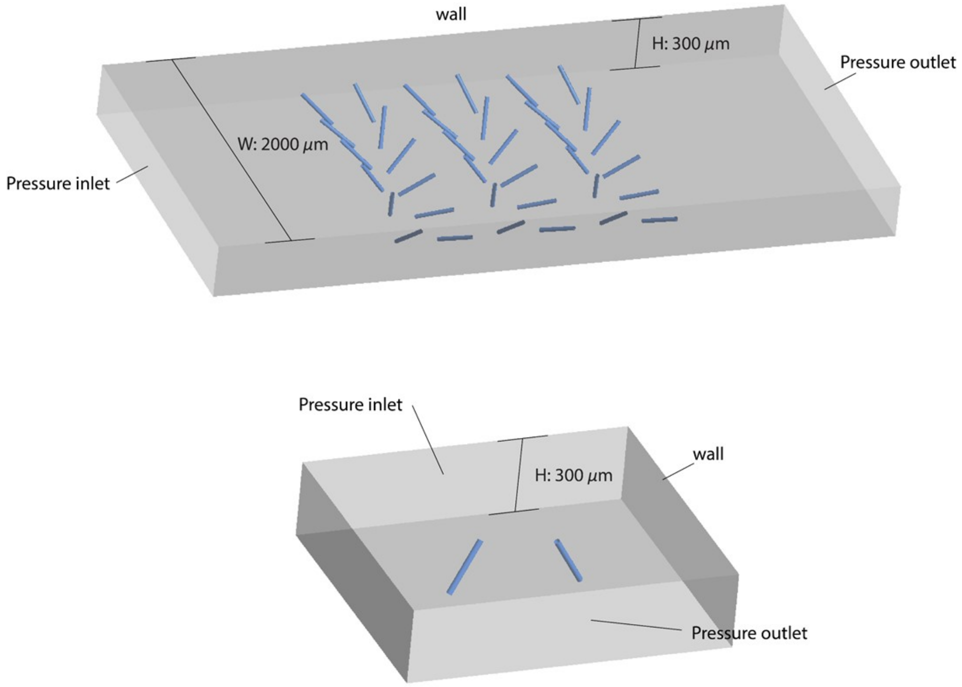

Figure S3: **Boundary conditions of Fluent model**

SM 4: Dimensionless comparison between magnetic, elastic and viscous effects

It is helpful to estimate the relative importance of different forces (elastic, magnetic and viscous) that influence the cilia dynamics. The elastic bending stiffness can be described as

$$EI = E\frac{\pi r^4}{4}, \qquad \text{(S4.1)}$$

with the cilia diameter d = 24 μm (r = 12 μm) and the elastic modulus E = 0.7 MPa,

$$EI = 1.14 \times 10^{-14}\, N \cdot m^2. \qquad \text{(S4.2)}$$

If we ignore the magnetic moment perpendicular to the cilia direction due to the shape induced anisotropy, the magnetic torque can be described as

$$\tau_{mag} = |m \times B| = \frac{(\mu_r - 1)V|B|^2 \sin 2\theta}{2\mu_0}, \qquad \text{(S4.3)}$$

where $\boldsymbol{m}$ is the induced magnetic moment along the length of the cilium, $\boldsymbol{B}$ is the external field (0.22 T) , μ_r is the relative permeability (1.32) of the cilia material as fitted from simulation and experimental data, V is the volume of the cilium (24 μm diameter, 240 μm long), and θ is the relative angle between $\boldsymbol{m}$ and $\boldsymbol{B}$. The largest torque can be applied when $\theta = 45^\circ$ and its value is:

$$\tau_{mag,max} = 6.7 \times 10^{-10}\, N \cdot m \qquad \text{(S4.4)}$$

One can estimate the minimum radius of curvature of the cilia close to the root as follows:

$$R_{root,min} = \frac{EI}{\tau_{mag,max}} = 1.7 \times 10^{-5} m, \qquad \text{(S4.5)}$$

which is about 14 times smaller than the length of cilia (240 μm), hence the magnetic field is sufficient to bend the cilia to a large angle.

The viscous drag force can be described as:

$$F_{drag} = f_d l = \frac{4\pi\mu v}{ln\left(\frac{l}{r}\right)} l/2, \qquad \text{(S5.6)}$$

where f_d is the coefficient for estimating the Stokes drag on the slender shaped cilia, considering the largest speed at the tip at 20 Hz (0.0023m/s), the viscous torque can be calculated as:

$$\tau_{vis} = F_{drag} l/2 = 1.8 \times 10^{-13}\, N \cdot m, \qquad \text{(S5.7)}$$

which is about three orders of magnitude smaller than the maximum magnetic torque. As a result, the cilia will closely follow the magnetic field with a small phase lag, which increases with frequency but will not significantly influence the motion trajectory at common frequencies (usually below 100 Hz).

Note that all three torques scale with l^3, meaning that if the aspect ratio of the cilia is kept constant, the relative motion of the cilia is largely unaffected by their overall sizes (until inertia starts to play a big role), which is another advantage of magnetic artificial cilia, especially at small length scales.

SM 5: Effect of z level on mixing effect

Fig. S4 shows the standard deviation of the concentration σ_c along the z-axis at different time points, from the simulation shown in Figure 5. Note that from equation (1), $MI_c = 0$ when $\sigma_c = 0.5$, and $MI_c = 1$ when $\sigma_c = 0$. It can be observed that over time, the region near the base of the cilia achieves effective mixing earlier, while the mixing in the upper region develops more slowly. This is as expected, since the tilted conical motion of the cilia creates more agitation of the fluids closer to the bottom than of the fluids closer to the top.

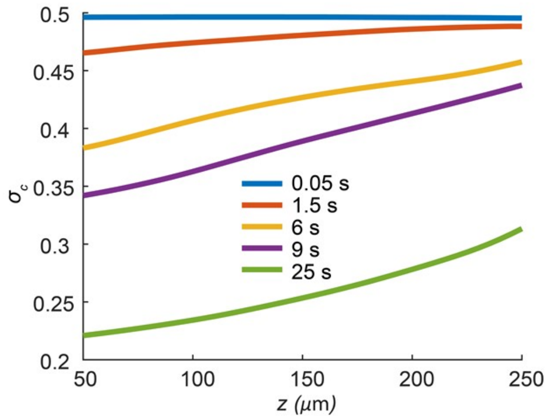

Figure S4: Standard deviation of the concentration σ_c at different planes along the z-axis at different time points, derived from the simulation shown in Figure 5 in the main text.

Movie S1.
The simulation result of fully coupled COMSOL model showing the cilia motion actuated by a rotating magnetic field with frequency of 1 Hz.

Movie S2.
The FLUENT simulation of mixing for double-cilia systems with the θ (phase) differences of 0°. The time of the video reflects the actual physical time. In the simulation, the actual frequency is approximately 19.5 Hz, influenced by the piecewise rotation velocity function and the time step. The visible cilia movement in the video does not reflect their true motion but is instead a result of the mismatch between the capture time points and the actual frequency.

Movie S3.
The FLUENT simulation of mixing for double-cilia systems with the θ (phase) differences of 90°. The time of the video reflects the actual physical time.

Movie S4.
The FLUENT simulation of mixing for double-cilia systems with the θ (phase) differences of 180°. The time of the video reflects the actual physical time.

Movie S5.
The experimental mixing result of the metachronal design at a cilia beating frequency of 20 Hz under anti-clockwise actuation. The camera captures images at a frame rate synchronized with the beating frequency. The video is sped up to three times the real-time speed.

Movie S6.
The experimental mixing result of the metachronal design at a cilia beating frequency of 20 Hz under clockwise actuation. The video is sped up to three times the real-time speed.

Movie S7.
The experimental mixing result of the reference design at a cilia beating frequency of 20 Hz under anti-clockwise actuation. The camera captures images at a frame rate synchronized with the beating frequency. The video is sped up to three times the real-time speed.

Movie S8.
The experimental mixing result of the reference design at a cilia beating frequency of 20 Hz under clockwise actuation. The video is sped up to three times the real-time speed.

Movie S9.
The FLUENT simulation result of the flow velocity vector during one beating cycle for the metachronal design.

Movie S10.

The FLUENT simulation result of the concentration evolution for the metachronal design.

Movie S11.

The experiment of mixing under flow conditions with a flow rate of 25 μL/min and a cilia beating frequency of 20 Hz. The camera captures images at a frame rate synchronized with the beating frequency.

Movie S12.

The experimental mixing result of the alternative design at a cilia beating frequency of 20 Hz under anti-clockwise actuation. The camera captures images at a frame rate synchronized with the beating frequency. The video is sped up to three times the real-time speed.

Movie S13.

The experimental mixing result of the alternative design at a cilia beating frequency of 20 Hz under clockwise actuation. The video is sped up to three times the real-time speed.
